# Macrophytes in Inland Waters: From Knowledge to Management (Second Volume)—New Contributions

**DOI:** 10.3390/plants15121847

**Published:** 2026-06-15

**Authors:** Angelo Troia

**Affiliations:** 1 Dipartimento di Scienze e Tecnologie Biologiche, Chimiche e Farmaceutiche (STeBiCeF), Università degli Studi di Palermo, 90123 Palermo, Italy; angelo.troia@unipa.it; 2 National Biodiversity Future Centre (NBFC), 90133 Palermo, Italy

## 1. Introduction

Inland waters—including rivers, lakes, wetlands, and groundwater-dependent ecosystems—are essential for global ecology and biodiversity. Although freshwater habitats cover less than 1% of the Earth’s surface, they support approximately 10% of all known species and nearly one-third of vertebrate species worldwide [1]. These ecosystems provide crucial ecological functions such as nutrient cycling, carbon storage, water purification, flood regulation, and climate regulation. Inland waters also sustain fisheries, agriculture, and drinking water supplies for billions of people worldwide [2].

Freshwater ecosystems are among the most threatened environments on the planet. Biodiversity loss in inland waters is occurring faster than in many terrestrial or marine ecosystems because of pollution, habitat degradation, river fragmentation, invasive species, overexploitation, and climate change [1]. Recent studies indicate that nearly one-quarter of freshwater species are currently at risk of extinction, highlighting the urgent need for integrated conservation and sustainable water management strategies [2].

Protecting inland waters is therefore fundamental not only for conserving biodiversity but also for maintaining ecosystem resilience, human well-being, and global environmental sustainability. Nature-based solutions, ecological restoration, and improved water governance are increasingly recognized as key tools to halt freshwater biodiversity decline and preserve ecosystem services at the global scale [3].

This second Special Issue follows the first one on the same topic [4], focusing on the “green” component, the strictly aquatic flora of inland waters (i.e., the plants living in the water or on its surface), that is generally poorly known despite its fundamental ecological role and the risk of extinction events to which it is subject.

## 2. Special Issue Contents

The nine articles—included in this second issue dedicated to “Macrophytes in Inland Waters: From Knowledge to Management”—collectively provide a comprehensive and multifaceted overview of current research on aquatic macrophytes, highlighting their importance across taxonomy, floristics, ecology, and applied environmental management. Together, they illustrate how these organisms serve not only as key components of freshwater ecosystems but also as valuable tools for understanding and managing environmental change.

From a taxonomic standpoint, Abdelahad et al. [5] emphasize the complexity and ongoing challenges in charophyte systematics. By reassessing a *Nitella* population from northern Italy, the authors reveal inconsistencies between morphological traits and type material, particularly regarding oospore ornamentation. This case highlights the limitations of traditional taxonomic approaches when type specimens are incomplete or ambiguous, and underscores the need for integrative studies combining morphology, molecular tools, and historical collections. The tentative identification of the Ferrara population as *Nitella axillaris* “sensu Auctores” also raises the issue of alien species introductions, linking taxonomy with biogeography and invasion biology.

At a broader spatial scale, Romanov et al. [6] provide a fundamental contribution to floristics by compiling the first comprehensive inventory of charophytes in the Caucasus. Their work integrates diverse data sources, including published research, herbarium collections, and field surveys, resulting in the identification of 27 species across six genera. Although the flora lacks endemic taxa and reflects general Palearctic patterns, its specific species composition gives it moderate regional distinctiveness. Importantly, the study highlights significant knowledge gaps, particularly the scarcity of recent data, and establishes a baseline for future research on distributional changes and conservation priorities.

Ecological investigations form a central component of these studies. Bellino and Baldantoni [7] examine charophyte communities in southern Italy, revealing low species diversity and a strong dependence on environmental conditions such as water chemistry, hydrology, and anthropogenic pressures. Their findings suggest that while some species, such as *Chara vulgaris*, are highly adaptable, others are more specialized and vulnerable, making them sensitive indicators of environmental change. Similarly, Zelnik et al. [8] analyze primary producer communities in a series of reservoirs along the Sava River (Slovenia), demonstrating how seasonal dynamics and water temperature strongly influence phytoplankton, phytobenthos, and macrophytes. Their results show that reservoir cascades can enhance phytoplankton growth and potentially promote harmful algal blooms, particularly under conditions of increased water retention time and temperature.

The role of macrophytes as bioindicators is further explored by Wrosz et al. [9], who use stable carbon and nitrogen isotope signatures in pondweed species to trace nutrient sources and anthropogenic impacts in Polish rivers and lakes. Their findings demonstrate clear differences between lotic and lentic systems, with higher δ15N values in rivers indicating stronger human influence, particularly from agriculture and wastewater inputs. This study highlights the capacity of aquatic plants to integrate environmental signals over time, providing a powerful tool for long-term ecosystem monitoring.

Biological invasions, mentioned in passing above [5], represent another important theme. Bučar et al. [10] investigate the distribution and ecological preferences of two invasive *Elodea* species in Croatia, showing how environmental variables such as nutrient levels, sediment type, and hydrology shape their distribution. The study reveals that *Elodea nuttallii* is particularly competitive in eutrophic and disturbed environments, where it can outcompete *E. canadensis* due to faster growth and greater tolerance to fluctuating conditions. These findings underline the importance of monitoring artificial and highly modified water bodies, which can act as hotspots for invasive species spread.

Several articles also explore applied and functional aspects of macrophyte ecology. Baibagyssov et al. [11] demonstrate the potential of combining remote sensing and machine learning techniques to estimate the biomass of *Phragmites australis* in the Syr Darya Delta (Kazakhstan). Their results reveal extensive reed coverage and high productivity, particularly in water-rich environments, highlighting both the ecological significance and economic potential of this species. At the same time, their study points to the need for long-term monitoring to account for environmental variability.

In a complementary perspective, Maranho and Gomez [12] review the adaptive traits that enable macrophytes to thrive in constructed wetlands used for wastewater treatment. They show that structural and physiological features, such as aerenchyma tissues and specialized root systems, allow these plants to tolerate harsh conditions while efficiently removing nutrients and pollutants. This makes macrophytes key components of sustainable water treatment systems, with additional benefits including habitat provision and carbon sequestration.

Finally, Van der Cruysse et al. [13] focus on restoration ecology, highlighting the potential of reintroducing native submerged macrophytes to improve stream biodiversity and ecosystem functioning. Their work emphasizes that successful restoration requires context-specific approaches, tailored to the particular pressures affecting each system. Submerged macrophytes can enhance water quality, stabilize habitats, and support diverse biological communities, making them valuable tools for achieving ecological objectives under frameworks such as the European Water Framework Directive.

Overall, these studies collectively demonstrate the central role of aquatic macrophytes (including charophytes) in freshwater ecosystems. They function as taxonomic challenges, indicators of environmental quality, drivers of ecological processes, and practical tools for monitoring, restoration, and management. By integrating different methodological approaches and spatial scales, this body of research highlights both the progress made and the challenges that remain in understanding and conserving aquatic plant diversity in a rapidly changing world.
